# Hay Fever—Its Cause, and Its Cure by Nux Vomica

**Published:** 1850-11

**Authors:** 


*Hay Fever—Its Cause, and its Cure by Nux Vomica.*—Hay Fever or Hay Asthma, is (in England) a well known complaint, by which many—especially females, and those of irritable surface—are annually distressed during the droughts of summer. Daily, or more frequent paroxysms of difficult respiration, severe prolonged sneezing, and a burning pain in the nostrils, eyes and face, are the usual symptoms. Dr. Craigie and others doubt the common explanation of the cause of the affection, viz., that it depends upon irritation of the peripheral extremities of the imperfectly protected nerves of the nostrils, etc., by the subtile pollen of the innumerable flowers, especially the grasses, which bloom in May and June, and with which the atmosphere must in some localities, be greatly charged. In the vicinity of hay fields and wild pasturage, if the weather be dry, those who are susceptible are certain to suffer; whereas, a rainy season, or sojourn on the sea-coast, will enable such persons to escape. Observation of many such facts has fully satisfied us, that there is no good ground for the incredulity expressed in the following passage by Dr. Craigie. "A particular variety," says he, "of catarrh, most prevalent in the summer months during the infloresence of the hay crop, in certain situations, has been believed to be connected with some irritative vapor exhaled from the flowers of some of the grasses, and has therefore been distinguished by the name of Hay Fever. It is doubtful whether this idea of the origin of the disorder be well founded; and it seems quite as likely that it is produced, as other varieties of catarrh, by imprudent exposure during excessive heat. The liberties which are often taken during extreme hot weather, are sufficient to induce catarrhal disorders, without having recourse to the assumption of a peculiar emanation." (*Practice of Physic*, vol. i, p. 823: Edin. 1847). We will venture to say, that no one who had seen such cases of Hay Fever as abound at this season, and as we have had to deal with during the present and former seasons, and who has carefully investigated into their personal and topographical peculiarities, could have written the paragraph just quoted. The popular theory is correct, that Hay Fever bears no relation whatever to common catarrh from exposure to cold. We feel assured that the cause and the cure in the two complaints are totally different.

We have been led to make these remarks, from having just perused a short note by Mr. G. T. Gream, in the *Lancet* of June 8th, 1850, p. 692, in which he reccommends Nux Vomica as a cure for this troublesome complaint. He correctly remarks, that the efficacy of this method is not generally known. The following is the essential part of Mr.

Gream's paper: "I am indebted to my friend Mr. Hammerton, of St. George's Hospital, for suggesting to me the nux vomica as a remedy in this complaint, which has frequently caused me, personally, much annoyance. It was administered by a friend of his to large numbers of the country people in his neighborhood, who flocked to him annually for relief, having experienced so much benefit from it. Having taken it for three years with decided effect, and having for nearly that time prescribed it for others, with equal success, I feel bound to publish through your columns, if you will do me the favor to insert this letter, the results of its use in a harassing disorder, with which many persons are at this time threatened. The preparation recommended, and which I have always prescribed, is the tincture of nux vomica of the "Dublin Pharmacopœia." Ten drops of this should be given for a dose, in water, and increased gradually to twenty drops, three times a day: the action of it should at first be watched. It is an agreeable light bitter; increases the appetite; and influences the Schneiderian membrane, no doubt through the medium of the nerves. I have accompanied the administration of the tincture with the application of an ointment (as high up in the nostrils as possible) composed of one drachm and a half of Goulard's extract, two ounces of spermaceti cerate, and a few drops of oil of roses or of bergamot.—*Lond. Journ. Med.*

